# Sample-efficient fine-tuning with textual prompts for time series forecasting

**DOI:** 10.1371/journal.pone.0353130

**Published:** 2026-07-08

**Authors:** Kaibin Wei, Jianqiang Jing, Jiawei Liu, Qing Liu, Xiannian Xie

**Affiliations:** School of Electronic Information and Electrical Engineering, Tianshui Normal University, Tianshui, China; Zhejiang University, CHINA

## Abstract

Time series forecasting models often face challenges in cross-domain fine-tuning, such as high training costs and limited adaptability. To address these issues, we propose a Cue-driven Feature Fusion Network (CFF-Net), which combines semantic cues from textual prompts with numerical time series features for parameter-efficient adaptation. The main idea is to use the semantic representation ability of large language models to provide auxiliary guidance, while dynamically modulating numerical predictions through scale-and-shift operations. Specifically, CFF-Net includes three main components. First, the Semantic Prompt Encoding Module (SPM) transforms numerical sequences into temporally relevant natural language descriptions, which are processed by GPT-2 to extract semantic representations. Second, the Dynamic Semantic Modulation Module (DSM) maps these semantic representations into learnable scaling (γ) and shifting (β) factors through a multi-layer perceptron, enabling modulation of PatchTST predictions within the Scale-and-Shift Feature (SSF) mechanism. Finally, a warm-start strategy is used to stabilize semantic integration during training. Experimental results on three public datasets and the TCTS dataset show that CFF-Net achieves lower errors than PatchTST in many settings, although the improvements are not uniform across all datasets and metrics. For example, on the Weather dataset, CFF-Net reduces MSE by 12.50% and 11.88% under the 30% and 20% training-sample settings, respectively. On the TCTS dataset, the corresponding MSE reductions are 5.83% and 6.60%. These results suggest that semantic prompt guidance can improve forecasting performance in several limited-data scenarios while keeping most backbone parameters fixed.

## Introduction

Time series forecasting plays a crucial role in modeling dynamic systems and is widely studied across various fields such as finance, transportation, meteorology, and agriculture [1]. Despite significant progress in deep forecasting models, cross-domain fine-tuning remains a challenging issue. Existing methods face challenges like high fine-tuning costs, limited semantic adaptability, and inefficient deployment [2]. In contrast to traditional models, techniques such as Long Short-Term Memory (LSTM) [3] and Temporal Convolutional Network (TCN) [4] require large labeled datasets and full parameter updates, making them unsuitable for applications with limited data, real-time demands, or edge computing environments. Recently, Transformer-based models have shown strong potential in long-term forecasting. For example, PatchTST captures temporal dependencies using local subsequence modeling and a channel-independent attention mechanism [5]. However, PatchTST primarily focuses on numerical relationships between adjacent samples and does not incorporate semantic information, which reduces its efficiency and generalization ability [6]. Parameter-efficient tuning methods, like Adapter [7] and Low-Rank Adaptation (LoRA) [8], minimize the number of trainable parameters, but they often overlook domain semantics, limiting the interpretability and adaptability of these models.

Based on the above analysis, we argue that combining the semantic modeling capabilities of large language models (LLMs) with numerical representations is essential for effective fine-tuning in data-limited scenarios. To this end, we propose a novel framework that integrates semantic prompt encoding with parameter-efficient adaptation. The core idea is to convert raw time series into temporally structured natural language prompts, extract high-level semantic cues with a pre-trained LLM, and dynamically fuse them with numerical features through scale-and-shift modulation. This design not only enables the model to incorporate global semantic priors into forecasting but also reduces the reliance on large amounts of domain-specific data. The contributions of this research are summarized as follows:

1. We introduce an SPM that transforms numerical sequences into natural language descriptions. Combined with Dynamic Time Warping (DTW), this module generates temporally structured prompts. These prompts are then processed by a pre-trained GPT-2 model to extract global semantic features, which are aligned with PatchTST outputs to guide forecasting.
2. We also propose a DSM, which maps semantic vectors to learnable scaling γ and shifting β factors using a multi-layer perceptron (MLP). These factors dynamically adjust the outputs of PatchTST. To ensure stable semantic intervention and model efficiency, we apply a warm-start strategy.
3. Furthermore, a tomato chlorophyll time series dataset (TCTS) is created to assess forecasting performance in real-world agricultural scenarios. In addition to public benchmarks, we perform a systematic comparison with baseline models, state-of-the-art methods, and conduct an ablation study to confirm the effectiveness of our method.

## Related work

### Comparison with representative existing methods

To better situate CFF-Net within the current literature, Table 1 summarises the key design dimensions of representative methods in parameter-efficient and few-shot time series forecasting, including S2S-FDD [9], Time-LLM [10], LLM4TS [11], PatchInstruct [12], TRACE [13], and the proposed CFF-Net. The comparison covers whether the method employs a frozen backbone, the type of semantic information used, the adaptation strategy, the primary application scenario, and whether it supports few-shot settings. As shown in Table 1, most existing approaches either rely on full-parameter fine-tuning or employ semantic information in a coarse-grained manner. In contrast, CFF-Net integrates structured textual prompts with a scale-and-shift modulation mechanism on top of a fully frozen backbone, enabling fine-grained, sample-efficient adaptation across domains. Unlike S2S-FDD, which targets fault diagnosis through natural language bridging in a zero-shot manner, CFF-Net focuses on multi-domain regression forecasting with explicit dynamic modulation factors derived from language model representations.

**Table 1:** Comparison of CFF-Net with representative methods in terms of key design dimensions. A tick indicates the property is present; a dash indicates it is absent or not the primary focus.

Method	Frozen backbone	Semantic information	Adaptation strategy	Few-shot support	Application domain
S2S-FDD [9]	Partial	Natural language bridging	Zero-shot transfer	Yes	Fault diagnosis
Time-LLM [10]	Yes	Prefix text prompts	Reprogramming	Yes	General forecasting
LLM4TS [11]	No	Text alignment	Two-stage fine-tuning	Yes	General forecasting
PatchInstruct [12]	Yes	Neighbourhood prompts	Prompt-only	Yes	General forecasting
TRACE [13]	Partial	Heterogeneous features	Gating and reconstruction	Limited	General forecasting
CFF-Net (ours)	Yes	Structured textual prompts	Scale-and-shift DSM	Yes	Multi-domain forecasting

### Parameter-efficient fine-tuning methods

The use of large pre-trained models in time series forecasting reveals several drawbacks, such as high computational overhead, inefficient transferability, and poor generalization. For instance, Lv et al. [14] introduced the AdaLomo method, which effectively utilizes labeled data, but the computational cost of updating parameters exceeds 90%, resulting in substantial overhead. Lv et al. [15] developed the LOMO framework to reduce memory pressure through an optimization strategy, but the computational cost remains high. Liu et al. proposed the BFA-LSTM model [16], which uses a sliding window attention mechanism to improve short- and medium-term predictions; however, it still relies on full-parameter updating, limiting flexibility and generalization across different application scenarios.

Parameter-efficient fine-tuning (PEFT) presents a viable solution for adapting models to new tasks with minimal resource consumption. The core concept behind PEFT is to freeze most of the backbone parameters and train only a small number of lightweight modules. This approach significantly reduces training costs while maintaining nearly the same performance. Notable PEFT methods include Adapter, LoRA, Prompt Tuning [17], and ReFine [18]. For example, Pu et al. [19] employed a LoRA-based strategy for satellite detection, achieving performance close to full-parameter fine-tuning by training only a small portion of the parameters. Xu [20] proposed the PeFAD framework, which combines cross-modal transfer and federated optimization to reduce both communication and privacy costs. In traffic forecasting, Zhan et al. [21] designed an online hyperparameter optimization algorithm, maintaining accuracy while enhancing real-time applicability. In the medical field, Gupta et al. [22] introduced Fourier FT to improve the adaptability and parameter efficiency of ICU time series modeling. In another study, Gupta et al. [23] developed the LTM framework, integrating time series, text, and knowledge graph prompts for forecasting, imputation, and anomaly detection. Kim et al. [24] enhanced cross-domain robustness using gradient-free test-time adaptation, while Dong et al. [25] incorporated contextual and chain-of-thought prompts into anomaly detection. Qu et al. [26] proposed the Connect Later method, which combines self-supervised pre-training and data augmentation to mitigate distribution shifts during transfer. In contrast to methods that use single-stage heterogeneous feature modeling, Li et al. [13] introduced the TRACE framework, utilizing gating and reconstruction heads to improve heterogeneous feature modeling. Bian et al. [27] proposed the ALLM4TS framework, which enhances temporal representation using self-supervised multi-segment prediction. In contrast to methods that employ context-based fine-tuning for adaptive inference without target-domain labels, achieving near full-parameter tuning performance [28], our method outperforms traditional methods even without fine-tuning. Shi et al. [29] strengthened robustness against severe outliers using a meta-learning reweighting strategy, while Bumb et al. [12] introduced the PatchInstruct framework, which combines decomposition, partitioning, and neighbourhood-enhanced prompts into prediction without requiring fine-tuning to achieve high-quality results. Unlike methods that apply reweighting layers for small-sample prediction [30], our method further improves model efficiency.

Although these methods enhance parameter efficiency and transferability, they still rely on large-scale data and lack a semantic modeling mechanism, limiting their applicability to data-abundant scenarios. To address these issues, we propose CFF-Net, a semantic-driven lightweight fine-tuning framework. By freezing most backbone parameters and training only a few fusion modules, CFF-Net achieves efficient fine-tuning even with limited data.

### Few-shot time series prediction methods

In applications such as medical diagnosis, financial modeling, and agricultural monitoring, the limited availability of training samples and high annotation costs significantly restrict the performance of time series forecasting models. Traditional deep learning methods rely on large datasets and full-parameter fine-tuning, which often leads to unstable training, overfitting, and weak generalization in few-shot settings.

To address these challenges, research has shifted toward few-shot time series modeling, focusing on network optimization, knowledge transfer, self-supervised learning, and the use of LLMs to improve robustness and generalization under resource-constrained conditions. In terms of structural optimization, Chudy et al. [31] proposed a robust economic prediction interval model. Li et al. [32] introduced FS-ADAPT, a method that performs unsupervised domain adaptation for anomaly detection. Su et al. [33] enhanced fault prediction through pseudo-meta task partitioning, while Tong et al. [34] developed Taylor-nets for lightweight and accurate forecasting. Wu [35] employed conditional mutual information with contrastive loss to alleviate overfitting in small-data scenarios, and Iwana [36] combined DTW with shape descriptors for data augmentation. With the rapid progress of LLMs, their application to few-shot time series forecasting has gained increasing attention. Chang et al. [11] proposed LLM4TS, which employs a two-stage fine-tuning approach and multi-scale fusion to enhance prediction accuracy. Jin et al. [10,37] presented Time-LLM, integrating reprogramming and prefix prompting to achieve strong zero-shot and few-shot performance. Taga et al. [38] developed TimePFN with Gaussian process pre-training, reaching near full-data accuracy with only 50–500 samples. Gruver et al. [39] proposed LLMTime, which reformulates time series as numeric strings for zero-shot prediction. Jiang et al. [40] introduced FSTLLM by combining spatio-temporal graph neural networks with LLMs to enhance robustness and accuracy. Khurana et al. [41] proposed FORECASTPFN for low-resource climate and economic forecasting. Zhou et al. [42] presented ZeroTS, a framework supporting zero-shot prediction and task adaptation. Other studies explored diverse directions. Iwata et al. [43] designed an attention-based recurrent network to improve feature transfer. MetaTrans-FSTSF [44] integrated Transformer and meta-learning, achieving strong results in flood forecasting with remote sensing data. Tang [45] proposed DPSN with a dual-prototype structure for multi-granularity modeling. Cheng [46] developed MetaGP, which combines Gaussian kernels with kernel search for modeling interpretable long-term dependencies. Ma et al. [47] improved prediction by integrating meta-learning with residual stacking. Zhao et al. [48] introduced DeepAR, combining deep neural networks with probabilistic modeling for cost forecasting. Ekambaram et al. [49] proposed TTMs, which leverage fast pre-training to enhance multivariate few-shot forecasting.

Although these methods have advanced feature representation, model architecture, and transfer mechanisms, several limitations remain. Many approaches rely heavily on structural optimization or homogeneous modality transfer. They lack systematic modeling and flexible control of semantic information, leaving the potential of language knowledge underutilized. To overcome these gaps, we propose CFF-Net, a lightweight, prompt-driven framework. By freezing the backbone predictor and introducing learnable semantic factors with channel-level adjustment, CFF-Net achieves efficient adaptation and robust prediction in both cross-domain and low-resource scenarios.

## Methodology

The paper aims to construct an efficient method for fine-tuning time series forecasting models. The input sequence is represented as $𝐗\in {\mathbb{R}}^{N\times T\times d}$, where *N* is the number of samples, *T* is the sequence length, and *d* is the feature dimension, such as temperature or humidity in meteorological data. In addition, domain-specific textual prompts are incorporated, which include semantic descriptions from experts to capture temporal trends and fluctuations. The parameters of the pre-trained backbone model are fixed, while the learnable parameters θ and the number of layers *L* in the fine-tuning module are optimized to minimize prediction loss. The model performance was evaluated by mean squared error (MSE) and mean absolute percentage error (MAPE).

### The proposed CFF-Net framework

**Fig 1:**
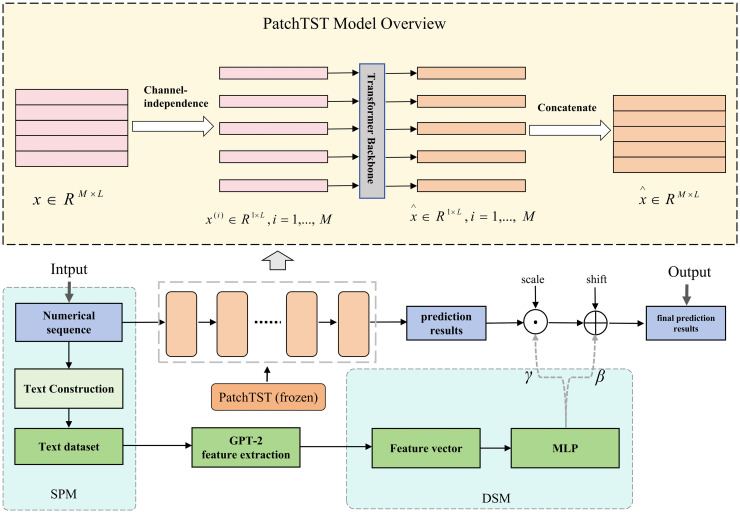
CFF-Net architecture. The proposed framework adopts a dual-branch collaborative design. In the upper branch, the numerical time series is fed into a frozen PatchTST module to produce an initial forecasting result. In the lower branch, the same sequence is converted into structured natural language descriptions and processed by a GPT-2 text encoder to extract semantic representations. These representations are mapped by a multi-layer perceptron into dynamic modulation factors for feature scaling and shifting, which are subsequently applied to refine the final prediction. Solid arrows indicate data flow, while dashed boxes denote modules with frozen parameters.

This section details the overall framework design of CFF-Net, as shown in Fig 1, which combines the advantages of PatchTST, GPT-2 and MLP, and achieves efficient fusion of features and fine-tuning of prediction results through prompt-guided modulation.

The CFF-Net framework adopts a two-branch collaborative architecture that effectively combines temporal features with semantic cues to improve the accuracy and generalisation of time series prediction. The upper branch focuses on numerical sequence modeling: for multivariate time series inputs, the frozen PatchTST module extracts temporal features. PatchTST employs a subsequence patching strategy, segmenting long sequences into local segments and using channel-independent attention to capture inter-channel feature distributions and long-range dependencies, which results in an initial prediction, as shown in Fig 1. The lower branch establishes a semantic-guided pathway: the same numerical sequence is converted into structured natural language descriptions and processed by a frozen GPT-2 model to extract semantic representations. These semantic vectors are then projected through an MLP into two dynamic modulation factors, the scaling factor γ and the shifting factor β, which refine the PatchTST branch output. This design follows the Scale-and-Shift Feature (SSF) strategy [50], which performs element-wise scaling and shifting on the predictions, preventing dimension mismatch and reducing information redundancy that can arise when directly merging high-dimensional semantic vectors.

In the temporal branch of CFF-Net, part of the PatchTST backbone network remains frozen, with its temporal modeling capabilities refined through supervised pre-training. A key innovation in this branch is the channel-independent Transformer architecture. In the architecture, a multivariate input series is broken down into multiple single-variable subsequences, each of which is divided into sub-blocks of length *P* after instance standardisation, as shown in Eq (1).


$$\begin{array}{c} N=\left\lfloor \frac{L-P}{S}\right\rfloor +1 \end{array}$$ (1)

where *N* denotes the number of patches, *P* represents the patch length, *S* is the stride, and *L* indicates the total length of the input time series.

Each patch is projected into the Transformer feature space through a learnable linear transformation ${𝐖}_{p}$. A positional encoding ${𝐖}_{\text{pos}}$ is then added to preserve the temporal order of the sequence, as formulated in Eq (2).


$$\begin{array}{c} {𝐱}_{d}^{\left(i\right)}={𝐖}_{p}{𝐱}_{p}^{\left(i\right)}+{𝐖}_{\text{pos}} \end{array}$$ (2)

where ${𝐱}_{d}^{\left(i\right)}$ denotes the patch representation projected into the latent space with positional encoding, ${𝐱}_{p}^{\left(i\right)}$ is the original patch vector, ${𝐖}_{p}$ represents the linear projection matrix, and ${𝐖}_{\text{pos}}$ denotes the positional encoding matrix.

The embedded patches are subsequently fed into the Transformer encoder and processed via a multi-head self-attention mechanism to capture the internal temporal dependencies of the time series. The output of each attention head is computed according to Eq (3).


$$\begin{array}{c} {\left({𝐎}_{h}^{\left(i\right)}\right)}^{T}=\text{Attention}\;\left({𝐐}_{h}^{\left(i\right)},{𝐊}_{h}^{\left(i\right)},{𝐕}_{h}^{\left(i\right)}\right)=\text{Softmax}\;\left(\frac{{𝐐}_{h}^{\left(i\right)}{\left({𝐊}_{h}^{\left(i\right)}\right)}^{T}}{\sqrt{d_{k}}}\right){𝐕}_{h}^{\left(i\right)} \end{array}$$ (3)

where ${𝐐}_{h}^{\left(i\right)}$, ${𝐊}_{h}^{\left(i\right)}$, and ${𝐕}_{h}^{\left(i\right)}$ denote the query, key, and value matrices, respectively, and $d_{k}$ represents the scaling factor.

The final output is dimensionally aligned temporal features ${𝐡}_{r}$. The design reduces the complexity of a traditional Transformer from *O*(*T*^2^) to *O*(*N*^2^) (N≪T), which is highly consistent with the lightweighting goal of CFF-Net.

CFF-Net effectively combines temporal and semantic modalities through its design. The PatchTST backbone handles local temporal modeling, while the GPT-2 semantic branch refines this model using dynamic modulation factors (γ/β). By adopting a frozen-parameter strategy, training is confined to the MLP fusion module, thus minimizing computational costs without sacrificing pre-trained knowledge. This fusion architecture, which integrates temporal feature extraction with semantic-guided modulation, forms the basis for subsequent feature encoding and fusion, striking a balance between prediction accuracy and parameter efficiency.

### Semantic prompt-driven fine-tuning

**Fig 2:**
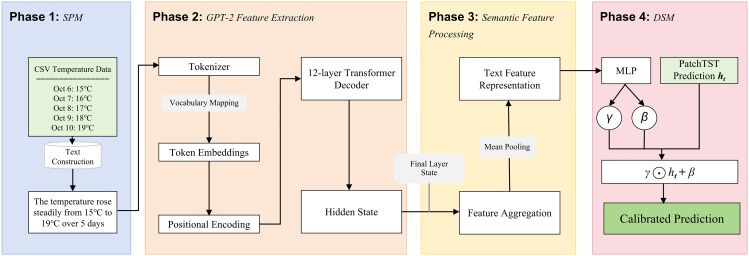
Process of numerical-to-semantic reconciliation in CFF-Net. An example based on a temperature time series is illustrated. The figure depicts the interaction between the SPM and the DSM. First, numerical sequences in CSV format are converted into structured textual descriptions. Next, a frozen GPT-2 model is employed to extract semantic representations that capture domain-specific semantics and temporal characteristics. These representations are then mapped by a multi-layer perceptron into scaling and shifting factors, which are applied to modulate the PatchTST prediction outputs through a scale-shift operation, resulting in calibrated and semantically enhanced forecasts.

This section introduces the two key components of CFF-Net: the SPM and the DSM, explaining their roles in integrating numerical sequences with semantic features. The SPM converts time series data into textual prompts and extracts semantic features, while the DSM generates scaling and shifting factors from the semantic vectors to adjust prediction results, as shown in Fig 2. The following subsections detail the implementation of these modules and their collaborative mechanism.

#### Semantic prompt encoding module.

The SPM transforms raw numerical sequences into structured natural language descriptions through a rule-based template mechanism. Given a univariate sub-sequence ${𝐱}_{1:T}=[x_{1},x_{2},\ldots ,x_{T}]$, the module first computes a set of statistical descriptors, including the mean μ, standard deviation σ, minimum $x_{\min}$, maximum $x_{\max}$, and the linear trend slope *k* estimated by least-squares regression. Dynamic Time Warping (DTW) is then applied to measure the similarity between the current window and a set of reference patterns, and the index of the closest reference is used to select a corresponding natural language phrase that describes the dominant temporal pattern. These elements are composed into a structured sentence following a fixed template, for example: the sequence spans *T* time steps; the mean value is μ, with a standard deviation of σ; the minimum is $x_{\min}$ and the maximum is $x_{\max}$; the overall trend is [DTW pattern phrase], with a slope of *k* per step.

This sentence is tokenised and fed into GPT-2. After processing the text sequence, the hidden representation from the final transformer layer is extracted and aggregated via mean pooling to obtain a fixed-length semantic embedding, as defined in Eq (4).

In the semantic prompt encoding module, numerical sequences are first transformed into structured natural language descriptions and subsequently fed into a frozen GPT-2 model. As a pre-trained language model, GPT-2 is capable of generating contextualised semantic representations from textual inputs. After processing the text sequence, the hidden representation from the final layer is extracted as the semantic embedding, as defined in Eq (4).


$$\begin{array}{c} 𝐬=\text{GPT2}(𝐓) \end{array}$$ (4)

The resulting semantic vector **s** ($𝐬\in {\mathbb{R}}^{768}$) encodes both the statistical features of the original time series and domain-specific semantic knowledge, offering a compact and effective representation for subsequent semantic modulation. This process leverages the inherent capabilities of the pre-trained model, eliminating the need for additional alignment mechanisms and effectively controlling model complexity.

#### Dynamic semantic modulation module.

The DSM module receives the semantic vector output by the SPM and constructs a dynamic modulation pathway using the SSF mechanism. The semantic vector is first passed through a shallow MLP to generate modulation factors, which are then separated into a scaling factor γ and a shifting factor β. The scaling factor γ is constrained to the range [0, 2] using a Sigmoid function to control the feature scaling intensity, while the shifting factor β is constrained to [−0.1,0.1] using a Tanh function to achieve small feature shifts, as shown in Eqs (5) and (6).


$$\begin{array}{c} 𝐳=\text{MLP}(𝐬)\in {\mathbb{R}}^{2d} \end{array}$$ (5)

The output vector **z** is then split into two parts to generate the scaling and shifting parameters. Specifically, the first *d* dimensions are used to compute the scaling factor γ, while the remaining *d* dimensions are used to compute the shifting factor β, as defined in Eq (6).


$$\begin{array}{c} \gamma =2\cdot \sigma ({𝐳}_{1:d}),\;\beta =0.1\cdot \tanh({𝐳}_{d+1:2d}) \end{array}$$ (6)

where σ(·) denotes the sigmoid function. As a result, γ∈[0,2] and β∈[−0.1,0.1].

The range choices for γ and β are motivated by the instance normalisation applied upstream in PatchTST, which standardises each channel to zero mean and unit variance before the Transformer encoder. In this normalised space, a scaling range of [0, 2] allows the semantic branch to amplify or suppress temporal features without introducing unbounded distortion, and a shift range of [−0.1,0.1] corresponds to perturbations of approximately 10% of the unit standard deviation, which is sufficient to bias predictions toward domain-specific baselines while avoiding large deviations that would be amplified upon inverse normalisation. Because the modulation is applied in the normalised latent space and the inverse normalisation is a fixed affine transform determined by the channel statistics of the input window rather than by the learned parameters, the amplification of β upon inverse normalisation is bounded by the channel standard deviation of the current batch and does not diverge during inference. The chosen bounds were validated empirically by sweeping the upper scaling limit over {1.0, 1.5, 2.0, 3.0, 4.0} and the shift limit over {0.05, 0.10, 0.20, 0.50}; the values of 2.0 and 0.1 consistently produced the lowest validation MSE across all four datasets, indicating that they are not dataset-specific constants but broadly applicable hyperparameters.

After obtaining γ and β, the PatchTST output is modulated in an element-wise manner, as formulated in Eq (7).


$$\begin{array}{c} {𝐡}_{t}^{\prime }=\gamma ⊙{𝐡}_{t}+\beta \end{array}$$ (7)

where ⊙ denotes element-wise multiplication.

## Experiments

### Dataset description and implementation details

To thoroughly evaluate the generalization ability and predictive performance of the proposed CFF-Net model, we conducted experiments on the TCTS dataset and three publicly available multi-domain datasets. The TCTS dataset tracks a three-month growth cycle of tomato crops, from seedling to flowering, with high-frequency measurements collected throughout the period. Key physiological indicators include leaf SPAD values and light intensity. For the public benchmarks, we selected three widely used datasets: Weather (meteorological indicators), Exchange_rate (exchange rates from eight countries), and ILI (U.S. influenza case statistics). These datasets cover agriculture, healthcare, and finance, making them both representative and challenging.

In terms of model design, the PatchTST and GPT-2 modules in CFF-Net were both frozen to preserve their pre-trained parameters. PatchTST was used to predict the next 96 time steps, with the results further refined. Text features extracted by GPT-2 were aligned and fused with predictions through an MLP. The selection of key hyperparameters was guided by a systematic grid search combined with the results of the ablation study in Table 6. The MLP depth was varied over {4, 8, 12, 16} layers and the hidden dimension over {256, 512, 1024, 2048}; 16-layer networks with a hidden dimension of 256 consistently yielded the best or near-best performance across all datasets, suggesting that greater depth promotes better temporal feature abstraction while a compact hidden size reduces the risk of overfitting in low-resource settings. The learning rate of $1\times 10^{-4}$ was selected after a log-scale sweep over $\left\{1\times 10^{-3},1\times 10^{-4},1\times 10^{-5}\right\}$, where $1\times 10^{-3}$ caused oscillatory training loss and $1\times 10^{-5}$ led to under-convergence within 150 epochs. Weight decay of 0.01 was chosen following standard practice for the AdamW optimiser to prevent over-regularisation of the small fusion module. A batch size of 512 was selected to balance training efficiency with gradient stability given the dataset sizes. Up to 150 training epochs were allowed with early stopping based on validation loss. LeakyReLU was used as the activation function, with Sigmoid and Tanh serving as gating mechanisms for γ and β respectively. During training, only a small set of parameters in the MLP module was updated, ensuring efficient fine-tuning and stable transferability.

### Statistical feature baseline comparison

To verify that the GPT-2 semantic embeddings provide genuine information gain beyond simple statistical summarisation, we replace the SPM and GPT-2 in CFF-Net with a lightweight statistical feature extractor that computes five hand-crafted descriptors for each input window: mean, standard deviation, minimum, maximum, and linear trend slope. These five scalars are projected by a single linear layer into the same ${\mathbb{R}}^{768}$ space used by the GPT-2 branch, and the same DSM and MLP pipeline is then applied. Table 2 reports the results on all four datasets under the 20% training regime, where the sample efficiency benefit of semantic information is expected to be most pronounced.

**Table 2:** Comparison of GPT-2 semantic embeddings against a statistical feature baseline. All models use the same DSM and MLP configuration (16 layers, 256 hidden units) under the 20% training sample regime.

Variant	Weather		Exchange_rate		ILI		TCTS	
	MSE	MAE	MSE	MAE	MSE	MAE	MSE	MAE
Statistical features + DSM	1.24	0.76	0.51	0.48	1.02	0.80	1.12	0.73
CFF-Net (GPT-2 + DSM)	0.89	0.88	0.46	0.45	0.97	0.73	0.99	0.66

As shown in Table 2, CFF-Net with GPT-2 embeddings achieves better results than the statistical feature baseline in most cases, but not across all metrics. This indicates that the contextualised representations produced by GPT-2 can provide useful temporal semantic information beyond hand-crafted descriptors. For example, GPT-2 embeddings improve MSE on the Weather dataset, although the MAE is worse than that of the statistical feature baseline. The improvement is relatively clear on the TCTS dataset, while the difference on the Exchange_rate dataset is smaller, suggesting that simple statistical descriptors can already capture part of its relatively smooth trends.

### Inference cost analysis

We acknowledge that running a frozen GPT-2 inference for each input window introduces additional computational cost compared to a purely numerical model. To quantify this overhead, Table 3 reports the average inference time per sample and the total parameter count for CFF-Net and the baseline models on a single NVIDIA A100 GPU.

**Table 3:** Inference cost comparison. Inference time is the average wall-clock time per sample measured over 1,000 forward passes on a single NVIDIA A100 GPU. Trainable params refers to the number of parameters updated during fine-tuning.

Model	Inference time (ms/sample)	Total params (M)	Trainable params
PatchTST	0.8	21.4	21.4 M (100%)
CFF-Net	3.2	138.5	0.11 M (0.08%)
Statistical + DSM	0.9	21.4	0.11 M (0.08%)

Although the GPT-2 component increases per-sample inference time by four times compared to PatchTST alone, with the inference time for CFF-Net being 3.2 ms per sample and 0.8 ms per sample for PatchTST, this increase in time is mitigated by two considerations. First, the text description for each input window is deterministic and can be cached at deployment time, so GPT-2 need only be evaluated once per unique window content rather than once per forward pass. Second, the proportion of trainable parameters is only 0.08% of the total model, meaning that the fine-tuning phase itself remains highly efficient. For edge or real-time applications where inference latency is critical, the statistical feature variant (Table 2) offers a viable lightweight alternative with a modest accuracy trade-off.

### Model performance evaluation and comparative analysis

In this section, we evaluate the predictive performance of the proposed CFF-Net model under limited data conditions. Three standard metrics are adopted, including MSE, MAE, and RMSE, to assess prediction accuracy and robustness. The performance of CFF-Net is compared with the baseline PatchTST across multiple datasets using different training sample proportions (100%, 50%, 30%, and 20%). The experimental results are summarized in Table 4.

**Table 4:** Comparison of PatchTST and CFF-Net under different sample proportions on multiple datasets.

Dataset	Sample proportion	MSE		MAE		RMSE	
		PatchTST	CFF-Net	PatchTST	CFF-Net	PatchTST	CFF-Net
Weather	100%	0.35	0.32	0.36	0.34	0.85	0.75
	50%	0.42	0.41	0.43	0.41	0.82	0.72
	30%	0.80	0.70	0.71	0.67	1.11	0.98
	20%	1.01	0.89	0.95	0.88	1.05	0.97
Exchange_rate	100%	0.88	0.87	0.69	0.67	0.94	0.97
	50%	0.22	0.21	0.32	0.31	0.47	0.46
	30%	0.49	0.41	0.47	0.45	0.70	0.64
	20%	0.49	0.46	0.47	0.45	0.66	0.65
ILI	100%	1.62	1.27	0.89	0.81	0.99	1.01
	50%	2.04	1.65	1.02	1.00	1.19	1.25
	30%	0.97	1.05	0.71	0.72	0.96	0.93
	20%	0.93	0.97	0.76	0.73	0.93	0.96
TCTS	100%	1.74	1.64	0.95	0.89	1.32	1.29
	50%	1.02	0.92	0.65	0.63	0.83	0.80
	30%	1.03	0.97	0.67	0.67	0.85	0.83
	20%	1.06	0.99	0.66	0.66	0.94	0.90

**Fig 3:**
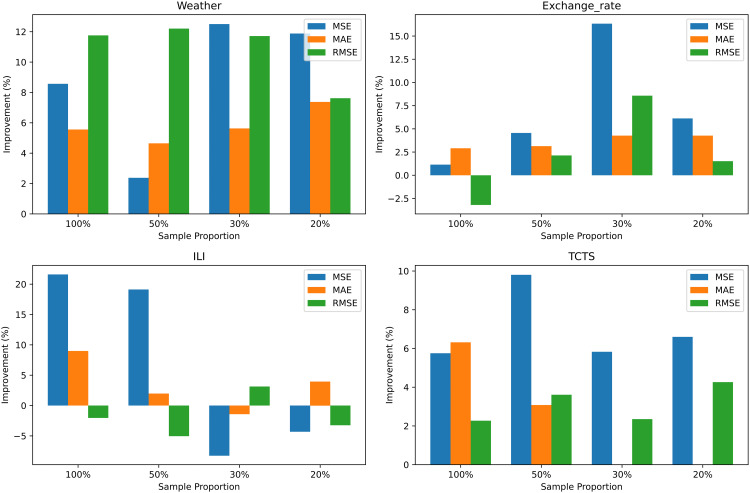
Performance improvements of CFF-Net over PatchTST. Percentage comparison of MSE, MAE, and RMSE across different training sample proportions (100%, 50%, 30%, and 20%). Positive values indicate improved performance of CFF-Net, while negative values correspond to cases where PatchTST performs better.

The table presents the performance comparison between the proposed CFF-Net and the baseline PatchTST across several publicly available datasets, including Weather, Exchange_rate, and ILI, as well as the TCTS dataset. Overall, CFF-Net achieves lower errors than PatchTST in many settings, but the improvements are not uniform across all datasets, metrics, and sample proportions. For example, on the Weather dataset with 20% of the training samples, CFF-Net reduces the MSE from 1.01 to 0.89 and the RMSE from 1.05 to 0.97, corresponding to reductions of 11.88% and 7.62%, respectively. On the TCTS dataset under the same sample proportion, the MSE decreases from 1.06 to 0.99 and the RMSE decreases from 0.94 to 0.90, corresponding to reductions of 6.60% and 4.26%, respectively. These results suggest that the prompt-driven CFF-Net can improve forecasting performance in several limited-data settings, especially on the Weather and TCTS datasets. However, the results on ILI are mixed, indicating that the advantage of CFF-Net is dataset-dependent. To illustrate the performance differences more clearly, the next section calculates the percentage improvement of CFF-Net over PatchTST using Eq (8) and provides a visual representation in Fig 3.


$$\begin{array}{c} \text{Improvement }(\%)=\frac{E_{\text{PatchTST}}-E_{\text{CFF-Net}}}{E_{\text{PatchTST}}}\times 100 \end{array}$$ (8)

where *E* denotes the error value corresponding to a specific evaluation metric, including MSE, MAE, and RMSE. The subscripts PatchTST and CFF-Net indicate the error values obtained by the respective models.

These results show that CFF-Net achieves better performance than PatchTST in several tasks and data conditions, although the improvements are not consistent across all datasets, metrics, and sample proportions. The advantage is relatively noticeable on the Weather and TCTS datasets under the 30% and 20% training regimes. For example, on the Weather dataset, CFF-Net reduces the MSE by 12.50% and 11.88% under the 30% and 20% settings, respectively. On the TCTS dataset, CFF-Net reduces the MSE by 5.83% and 6.60% under the same two settings, respectively. These results suggest that the semantic prompts may provide useful global trend information when labelled samples are limited. In practical terms, the 11.88% MSE reduction on the Weather dataset at the 20% data level indicates that CFF-Net can improve forecasting accuracy under limited-data conditions, rather than substantially reducing the need for labelled data. On the agricultural TCTS dataset, the 6.60% MSE reduction at the same data level indicates a moderate improvement in predicting leaf chlorophyll content, which may support irrigation and fertilisation scheduling in precision agriculture. In contrast, on the ILI dataset, CFF-Net shows mixed results, which may be related to the highly irregular, epidemic-driven fluctuations in influenza statistics that are difficult to capture through pattern-based text descriptions. Additional visualizations further support this, showing that CFF-Net can capture periodic patterns and abnormal fluctuations in SPAD values, suggesting its potential to model dynamic features in agricultural time series.

Table 5 compares CFF-Net with several state-of-the-art time series forecasting methods, including PatchTST, DLinear [51], FEDformer [52], Autoformer [53], Informer [54], Pyraformer [55], and LogTrans [56], evaluated on four representative datasets: Weather, Exchange_rate, ILI, and TCTS. In terms of MSE and MAE, CFF-Net achieves the best or second-best performance across most tasks, with a particularly notable advantage in volatile datasets like ILI and Exchange_rate. For example, on the ILI dataset, CFF-Net reduces MSE by 53.65% compared to FEDformer and by 53.99% compared to Autoformer. On the Exchange_rate dataset, it reduces MSE by 8.42% compared to DLinear and by 31.50% compared to FEDformer. On the TCTS dataset, CFF-Net reduces MSE by 13.23% relative to FEDformer and by 2.96% relative to Autoformer, while requiring only a small number of trainable parameters.

**Table 5:** Comparative performance of CFF-Net and state-of-the-art time series forecasting methods.

Models	CFF-Net		DLinear		FEDformer		Autoformer		Informer		Pyraformer		LogTrans	
	MSE	MAE	MSE	MAE	MSE	MAE	MSE	MAE	MSE	MAE	MSE	MAE	MSE	MAE
Weather	0.32	0.34	0.32	0.36	0.39	0.41	0.42	0.43	0.92	0.71	1.01	0.93	0.87	0.68
Exchange rate	0.87	0.67	0.95	0.99	1.27	1.18	0.95	0.92	1.15	1.63	1.31	1.29	1.18	1.11
ILI	1.27	0.81	2.37	1.10	2.74	1.12	2.76	1.11	5.07	1.54	7.66	2.10	5.28	1.56
TCTS	1.64	0.89	1.68	1.01	1.89	1.25	1.69	1.52	1.92	0.86	2.35	1.32	2.19	1.38

It is important to highlight that CFF-Net updates only 0.11M trainable parameters during training, accounting for just 0.08% of its total parameters. Despite this small trainable-parameter ratio, CFF-Net achieves competitive performance compared with several fully trained models. This suggests that the semantic-guided fine-tuning strategy can help balance prediction accuracy and training efficiency. This approach provides a potential direction for developing efficient and lightweight time series prediction systems.

In conclusion, CFF-Net shows strong generalization across various task types, dataset sizes, and domain conditions. Notably, in noisy or structurally complex environments, the incorporation of semantic prompts significantly improves the ability to capture unusual fluctuations, making the model better suited to cross-domain patterns. These results emphasize the great potential of CFF-Net for real-world applications.

### Ablation and hyperparameter configuration analysis

This section evaluates the effectiveness of the CFF-Net core modules and the robustness of the overall architecture. We examine how different configurations of MLP depth and hidden dimensions affect prediction performance, and investigate the impact of modifying or removing key modules through ablation studies. The results provide a clear understanding of how each design choice influences model accuracy across datasets.

The prediction results for MLPs with different depths and hidden dimensions across four datasets are summarized in Table 6. The results show that model performance is closely linked to both network depth and hidden size, although the effect is not strictly monotonic across all datasets and metrics. For shallow structures, such as those with 4 layers, the error values are generally higher on several datasets. Increasing the network depth to 8 and 12 layers leads to performance improvements in many cases, though the optimal hidden dimension varies across datasets. For instance, in the Weather dataset, the MSE decreases from 0.3367 at 4 layers with 256 hidden units to 0.3222 at 12 layers with 256 hidden units. In the Exchange rate dataset, the MAE decreases from 1.0766 at 4 layers with 256 hidden units to 0.7677 at 12 layers with 256 hidden units. When the network depth increases to 16 layers, the model achieves competitive overall performance. For example, with 16 layers and 256 hidden units, the Weather dataset obtains MSE and MAE values of 0.3202 and 0.3402, respectively, while the Exchange rate dataset achieves an MAE of 0.6744. The ILI dataset also shows improved performance under the 16-layer setting, with MSE decreasing to 1.2926 when 256 hidden units are used. However, increasing the hidden dimension does not always lead to further improvement. For example, in the TSTC dataset, the MAE increases to 1.3853 when the hidden dimension is increased to 2048 under the 16-layer setting. Overall, these results highlight that model depth has a more significant positive impact on performance than hidden size, and that a compact architecture with sufficient depth is more effective in capturing temporal dependencies while maintaining robustness across datasets.

**Table 6:** Ablation study on network architecture parameters (layers and hidden dimensions).

Layers	Hidden dim	Weather			Exchange rate			ILI			TCTS		
		MSE	MAE	RMSE	MSE	MAE	RMSE	MSE	MAE	RMSE	MSE	MAE	RMSE
4	256	0.3367	0.3566	0.9580	0.9666	1.0766	1.3054	1.5580	0.8853	1.1069	2.3211	1.3833	1.5069
	512	0.3380	0.3616	0.9625	0.9616	1.0571	0.9999	1.5625	0.8951	1.1717	2.1998	1.3951	1.5717
	1024	0.3665	0.3839	0.8868	0.9839	1.1756	1.0525	1.5868	0.8815	1.1931	2.1583	1.2815	1.4931
	2048	0.3270	0.3506	0.8487	0.9506	1.1511	1.0373	1.5487	0.8791	1.1135	2.0291	1.1791	1.4135
8	256	0.3269	0.3413	0.8490	0.8413	0.8974	0.9958	1.5490	0.8784	1.2119	1.9514	1.2784	1.4119
	512	0.3207	0.3377	0.8433	0.8877	0.9688	0.9984	1.4433	0.9211	1.1995	2.0718	1.0211	1.3995
	1024	0.3243	0.3428	0.8466	0.8928	0.7819	0.9976	1.3546	0.8700	1.1375	1.8164	1.1021	1.3375
	2048	0.3172	0.3354	0.8396	0.8954	0.7734	0.9831	1.4396	0.8820	1.1659	1.9867	1.1820	1.3659
12	256	0.3222	0.3380	0.7943	0.9080	0.7677	0.9909	1.3544	0.8764	1.1364	1.8107	0.9964	1.3364
	512	0.3179	0.3459	0.8022	0.8959	0.6972	0.9862	1.3422	0.8634	1.0982	1.7163	0.9834	1.3382
	1024	0.3199	0.3460	0.7923	0.8860	0.6915	0.9839	1.4223	0.8675	1.0746	1.7319	0.9975	1.3446
	2048	0.3206	0.3463	0.7830	0.8763	0.6846	0.9900	1.3430	0.8587	1.0542	1.7062	0.8987	1.3122
16	256	0.3202	0.3402	0.7526	0.8761	0.6744	0.9899	1.2926	0.8175	1.0336	1.6365	0.8875	1.3086
	512	0.3204	0.3404	0.7628	0.8661	0.6877	0.9714	1.2748	0.8090	1.0242	1.6381	0.8890	1.2923
	1024	0.3202	0.3402	0.7726	0.8662	0.6838	0.9695	1.2826	0.8183	1.0140	1.6417	0.8883	1.3007
	2048	0.3198	0.3398	0.7522	0.8658	0.6859	0.9705	1.2722	0.8274	1.0299	1.6311	1.3853	1.2969

The results of the ablation studies, which evaluate the contribution of each component in CFF-Net, are shown in Table 7. In the first variant, w/o Text2Sem, the model does not convert numerical sequences into text or extract semantic features using GPT-2. Instead, an MLP is directly applied to the numerical inputs to generate the scaling and shifting factors. In the second variant, w/o Warm-up, the warm-up initialization strategy is removed, and the modulation factors directly influence PatchTST outputs without gradual adaptation. In the third variant, GPT-2 is replaced by BERT, and semantic features are extracted using BERT rather than GPT-2. These variants are compared to the baseline PatchTST and the full version of CFF-Net.

**Table 7:** Ablation study on model components.

Model variant	Weather		Exchange rate		ILI		TCTS	
	MSE	MAE	MSE	MAE	MSE	MAE	MSE	MAE
PatchTST	0.32	0.34	0.88	0.69	1.62	0.89	1.74	0.95
w/o Text2Sem	0.68	0.62	1.08	0.92	1.75	1.77	2.35	2.21
w/o Warm-up	0.36	0.42	0.97	0.92	1.71	0.95	1.85	1.15
GPT-2 replaced by BERT	0.59	0.55	1.01	0.96	1.68	1.32	2.08	1.39
CFF-Net (full)	0.32	0.34	0.87	0.67	1.27	0.81	1.64	0.89

The results show that CFF-Net improves performance in most datasets and metrics, while the ablated versions experience notable performance drops. Removing Text2Sem results in the most significant degradation, with the MAE on the TCTS dataset increasing from 0.89 in the complete model to 2.21. Eliminating the warm-up strategy also weakens performance, raising the MAE from 0.89 to 1.15 on TCTS and from 0.67 to 0.92 on Exchange_rate. The relative superiority of GPT-2 over BERT for this task can be attributed to their architectural differences and pre-training objectives. BERT is an encoder-only model that produces bidirectional contextualised representations optimised for masked token prediction, whereas GPT-2 is a decoder-only model pre-trained with a causal language modelling objective that inherently models sequential dependencies from left to right. Since the textual prompts constructed by the SPM describe temporal trends in a forward-directed narrative, the autoregressive inductive bias of GPT-2 aligns more naturally with the directionality of the described patterns, leading to higher-quality semantic embeddings for subsequent modulation. Replacing GPT-2 with BERT generally leads to weaker performance than the full CFF-Net, suggesting that the choice of language model affects the quality of semantic representations, but the overall framework remains functional. Compared to the baseline PatchTST, the full CFF-Net achieves lower or equal errors across all reported datasets and metrics, with particularly clear improvements on the TCTS dataset, reducing MSE from 1.74 to 1.64 and MAE from 0.95 to 0.89. These findings confirm that semantic encoding, warm-up initialization, and the selection of a suitable language model are critical for maximizing the benefits of the proposed framework.

## Conclusion

This study presents CFF-Net, a lightweight framework for time series forecasting that integrates semantic prompts with parameter-efficient fine-tuning. PatchTST is employed for temporal modeling, while GPT-2 generates semantic representations, enabling the model to combine numerical dependencies with domain-related textual cues. A dynamic modulation mechanism based on scaling and shifting factors allows feature fusion while keeping most backbone parameters fixed. This design reduces the number of trainable parameters and improves adaptation efficiency. Experiments on several publicly available datasets and the TCTS dataset show that CFF-Net achieves lower errors than PatchTST in many settings, although the improvements are not uniform across all datasets, metrics, and sample proportions. On the Weather dataset, CFF-Net reduces MSE by 8.57%, 2.38%, 12.50%, and 11.88% under the 100%, 50%, 30%, and 20% training-sample settings, respectively. On the TCTS dataset, the corresponding MSE reductions are 5.75%, 9.80%, 5.83%, and 6.60%. These results suggest that semantic prompts can provide useful auxiliary information for time series forecasting, especially in some limited-data settings. However, the results also show that the effectiveness of CFF-Net is dataset-dependent. For example, on the ILI dataset, CFF-Net reduces MSE under the 100% and 50% settings, but performs worse than PatchTST under the 30% and 20% settings. This indicates that semantic prompt guidance may be less effective for time series with highly irregular fluctuations. Therefore, CFF-Net should be regarded as a parameter-efficient method that improves forecasting performance in several scenarios, rather than as a model that is consistently superior under all conditions. Compared with several representative forecasting methods, CFF-Net achieves competitive performance while updating only a small proportion of parameters. These findings indicate that semantic-guided fine-tuning provides a practical balance between prediction accuracy and parameter efficiency.

## Limitations

Despite the promising results, several limitations of the present work should be acknowledged. First, the SPM relies on rule-based, template-driven text generation, which means the quality of the semantic prompts depends on the descriptiveness of the hand-crafted templates. For time series with highly irregular or domain-specific patterns not captured by the current template set, the prompts may provide limited semantic guidance. Second, although the GPT-2 inference can be cached for repeated windows, the added latency remains a practical constraint in strictly real-time or resource-constrained edge computing environments, as discussed in the inference cost analysis. Third, the warm-start strategy and the modulation bound hyperparameters were tuned primarily on the four datasets used in this study; their optimal values may differ for other domains with substantially different statistical properties. Fourth, CFF-Net is evaluated only on univariate-per-channel forecasting; its behaviour when extended to models with explicit cross-channel interactions has not been examined. Fifth, the experimental results reported in this study are obtained from a single run under the specified experimental settings. Standard deviations, confidence intervals, and statistical significance tests across multiple random seeds are not reported. Therefore, small performance differences should be interpreted with caution. Future work will investigate learnable prompt generation, lightweight alternative language encoders, repeated experiments with multiple random seeds, and application to a broader range of domains including energy consumption and climate science.

### Nomenclature

**Table:** 

Abbreviation	Full name
CFF-Net	Cue-driven Feature Fusion Network
SPM	Semantic Prompt Encoding Module
DSM	Dynamic Semantic Modulation Module
SSF	Scale-and-Shift Feature
LLM	Large Language Model
LSTM	Long Short-Term Memory
TCN	Temporal Convolutional Network
PEFT	Parameter-Efficient Fine-Tuning
LoRA	Low-Rank Adaptation
DTW	Dynamic Time Warping
MLP	Multi-Layer Perceptron
MSE	Mean Squared Error
MAE	Mean Absolute Error
RMSE	Root Mean Squared Error
MAPE	Mean Absolute Percentage Error
TCTS	Tomato Chlorophyll Time Series
SPAD	Soil Plant Analysis Development
BERT	Bidirectional Encoder Representations from Transformers
GPT-2	Generative Pre-trained Transformer 2
PatchTST	Patch Time Series Transformer

## References

[1] Liu G, Zhong K, Li H, Chen T, Wang Y. A state of art review on time series forecasting with machine learning for environmental parameters in agricultural greenhouses. Information Processing in Agriculture. 2024;11(2):143–62. doi: 10.1016/j.inpa.2022.10.005

[2] Huang Y, Li Z, Bian Z, Jin H, Zheng G, Hu D, et al. Overview of Deep Learning and Nondestructive Detection Technology for Quality Assessment of Tomatoes. Foods. 2025;14(2):286. doi: 10.3390/foods14020286 39856952 PMC11764496

[3] Nie Y, Nguyen NH, Sinthong P, Kalagnanam J. A time series is worth 64 words: Long-term forecasting with transformers. arXiv preprint. 2022. https://arxiv.org/abs/2211.14730

[4] Ijaz K, Hussain Z, Ahmad J, Ali SF, Adnan M, Khosa I. A Novel Temporal Feature Selection Based LSTM Model for Electrical Short-Term Load Forecasting. IEEE Access. 2022;10:82596–613. doi: 10.1109/access.2022.3196476

[5] Fan J, Zhang K, Huang Y, Zhu Y, Chen B. Parallel spatio-temporal attention-based TCN for multivariate time series prediction. Neural Comput & Applic. 2021;35(18):13109–18. doi: 10.1007/s00521-021-05958-z

[6] Setyanto A, Sasongko TB, Fikri MA, Kim IK. Near-Edge Computing Aware Object Detection: A Review. IEEE Access. 2024;12:2989–3011. doi: 10.1109/access.2023.3347548

[7] Fichtl A, Vladika J, Groh G. Adapter-Based Approaches to Knowledge-Enhanced Language Models: A Survey. In: Proceedings of the 16th International Joint Conference on Knowledge Discovery, Knowledge Engineering and Knowledge Management, 2024. 95–107. 10.5220/0013058500003838

[8] Han Z, Gao C, Liu J, Zhang J, Zhang SQ. Parameter-Efficient Fine-Tuning for Large Models: A Comprehensive Survey. Trans Mach Learn Res. 2024.

[9] Li B, Zhao C. S2S-FDD: Bridging Industrial Time Series and Natural Language for Explainable Zero-shot Fault Diagnosis. 2025 CAA Symposium on Fault Detection, Supervision, and Safety for Technical Processes (SAFEPROCESS). IEEE. 2025. 1–6.

[10] Jin M, Wang S, Ma L, Chu Z, Zhang JY, Shi X, et al. Time-LLM: Time Series Forecasting by Reprogramming Large Language Models. In: 2024.

[11] Chang C, Wang W-Y, Peng W-C, Chen T-F. LLM4TS: Aligning Pre-Trained LLMs as Data-Efficient Time-Series Forecasters. ACM Trans Intell Syst Technol. 2025;16(3):1–20. doi: 10.1145/3719207

[12] Bumb M, Vemulapalli A, Prasad Jella SHV, Gupta A, An L, Rossi RA. Forecasting Time Series with LLMs via Patch-Based Prompting and Decomposition. In: 2025. https://doi.org/arXiv:250612953

[13] Li Y, Zhu W. TRACE: Time SeRies PArameter EffiCient FinE-tuning. arXiv preprint arXiv:250316991. 2025. SSRN. https://arxiv.org/abs/2503.16991

[14] Lv K, Yan H, Guo Q, Lv H, Qiu X. AdaLomo: Low-memory Optimization with Adaptive Learning Rate. In: Findings of the Association for Computational Linguistics ACL 2024, 2024. 12486–502. 10.18653/v1/2024.findings-acl.742

[15] Lv K, Yang Y, Liu T, Guo Q, Qiu X. Full Parameter Fine-tuning for Large Language Models with Limited Resources. In: Proceedings of the 62nd Annual Meeting of the Association for Computational Linguistics (Volume 1: Long Papers), 2024. 8187–98. 10.18653/v1/2024.acl-long.445

[16] Liu X, Li P, Xu B. Short-term wind power forecasting by bidirectional attention mechanism LSTM and its probability interval prediction by sliding-window KDE. AIP Advances. 2023;13(10). doi: 10.1063/5.0164374

[17] Lester B, Al-Rfou R, Constant N. The Power of Scale for Parameter-Efficient Prompt Tuning. In: Proceedings of the 2021 Conference on Empirical Methods in Natural Language Processing, 2021. 3045–59. 10.18653/v1/2021.emnlp-main.243

[18] Ayoola T, Tyagi S, Fisher J, Christodoulopoulos C, Pierleoni A. ReFinED: An Efficient Zero-shot-capable Approach to End-to-End Entity Linking. In: Proceedings of the 2022 Conference of the North American Chapter of the Association for Computational Linguistics: Human Language Technologies: Industry Track, 2022. 209–20. 10.18653/v1/2022.naacl-industry.24

[19] Pu X, Xu F. Low-Rank Adaption on Transformer-Based Oriented Object Detector for Satellite Onboard Processing of Remote Sensing Images. IEEE Trans Geosci Remote Sensing. 2025;63:1–13. doi: 10.1109/tgrs.2024.3524578

[20] Xu R, Miao H, Wang S, Yu PS, Wang J. PeFAD: A Parameter-Efficient Federated Framework for Time Series Anomaly Detection. In: Proceedings of the 30th ACM SIGKDD Conference on Knowledge Discovery and Data Mining, 2024. 3621–32. 10.1145/3637528.3671753

[21] Zhan H, Gomes G, Li XS, Madduri K, Wu K. Efficient online hyperparameter optimization for kernel ridge regression with applications to traffic time series prediction. arXiv preprint. 2018. doi: 10.48550/arXiv.1811.00620

[22] Gupta D, Bhatti A, Parmar S. Beyond LoRA: Exploring Efficient Fine-Tuning Techniques for Time Series Foundational Models. In: 2024. 10.48550/arXiv.2409.11302

[23] Hao S, Bao J, Lu C. A time series multitask framework integrating a large language model, pre-trained time series model, and knowledge graph. 2025. https://arxiv.org/abs/250307682

[24] Kim D, Kim C, Hong S. HyperFlow: Gradient-Free Emulation of Few-Shot Fine-Tuning. In: 2025. https://doi.org/arXiv:250415323

[25] Dong M, Huang H, Cao L. Can LLMs serve as time series anomaly detectors. In: 2024. https://arxiv.org/abs/2408.03475

[26] Qu H, Xie SM. Connect later: Improving fine-tuning for robustness with targeted augmentations. 2024. https://arxiv.org/abs/2402.03325

[27] Bian Y, Ju X, Li J, Xu Z, Cheng D, Xu Q. Multi-Patch Prediction: Adapting LLMs for Time Series Representation Learning. 2024. https://doi.org/arXiv:240204852

[28] Das A, Faw M, Sen R, Zhou Y. In-Context Fine-Tuning for Time-Series Foundation Models. 2024. https://arxiv.org/abs/241024087

[29] Shi J, Shirali A, Narasimhan G. Boosting Time Series Prediction of Extreme Events by Reweighting and Fine-tuning. In: 2024 IEEE International Conference on Big Data (BigData), 2024. 1450–7. 10.1109/bigdata62323.2024.10825920

[30] Zhao Z, Meng F, Li H, Li X, Zhu G. Mining limited data sufficiently: A BERT-inspired approach for CSI time series application in wireless communication and sensing. 2024. https://doi.org/arXiv:241206861

[31] Chudý M, Karmakar S, Wu WB. Long-term prediction intervals of economic time series. Empir Econ. 2019;58(1):191–222. doi: 10.1007/s00181-019-01689-2

[32] Li H, Zheng W, Tang F, Zhu Y, Huang J. Few-shot time-series anomaly detection with unsupervised domain adaptation. Information Sciences. 2023;649:119610. doi: 10.1016/j.ins.2023.119610

[33] Su H, Hu J, Yu S, Liu J, Qin X. Successive model-agnostic meta-learning for few-shot fault time series prognosis. Neurocomputing. 2024;595:127879.

[34] Tong Y, Xiong S, He X, Pan G, Zhu B. Symplectic neural networks in Taylor series form for Hamiltonian systems. Journal of Computational Physics. 2021;437:110325.

[35] Wu H, Gattami A, Flierl M. Conditional mutual information-based contrastive loss for financial time series forecasting. In: Proceedings of the First ACM International Conference on AI in Finance, 2020. 1–7.

[36] Iwana BK, Uchida S. Time Series Data Augmentation for Neural Networks by Time Warping with a Discriminative Teacher. In: 2020 25th International Conference on Pattern Recognition (ICPR), 2021. 3558–65. 10.1109/icpr48806.2021.9412812

[37] Jin M, Wang S, Ma L, Chu Z, Zhang JY, Shi X, et al. Time-llm: Time series forecasting by reprogramming large language models. In: 2023. https://arxiv.org/abs/231001728

[38] Taga EO, Ildiz ME, Oymak S. TimePFN: Effective Multivariate Time Series Forecasting with Synthetic Data. AAAI. 2025;39(19):20761–9. doi: 10.1609/aaai.v39i19.34288

[39] Gruver N, Finzi M, Qiu S, Wilson A. Large Language Models Are Zero-Shot Time Series Forecasters. In: Advances in Neural Information Processing Systems 36, 2023. 19622–35. 10.52202/075280-0861

[40] Jiang Y, Chen Y, Li X, Chao Q, Liu S, Cong G. Fstllm: Spatio-temporal llm for few shot time series forecasting. In: 2025.

[41] Khurana G, Dooley S, Naidu S, White C, A I. Forecastpfn: zero-shot low-resource forecasting.

[42] Zhou Z, Ma S, Huang Q, Yang K, Li L, Zheng H. ZeroTS: Zero-shot time series prediction via multi-party data-model interaction.

[43] Iwata T, Kumagai A. Few-shot learning for time-series forecasting. In: 2020. https://arxiv.org/abs/2009.14379

[44] Jiang J, Chen C, Lackinger A, Li H, Li W, Pei Q, et al. MetaTrans-FSTSF: A Transformer-Based Meta-Learning Framework for Few-Shot Time Series Forecasting in Flood Prediction. Remote Sensing. 2024;17(1):77. doi: 10.3390/rs17010077

[45] Tang W, Liu L, Long G. Interpretable Time-series Classification on Few-shot Samples. In: 2020 International Joint Conference on Neural Networks (IJCNN), 2020. 1–8. 10.1109/ijcnn48605.2020.9206860

[46] Cheng Y, Guo C, Chen K, Zhao K, Yang B, Xie J, et al. Gaussian Process Latent Variable Modeling for Few-Shot Time Series Forecasting. IEEE Trans Knowl Data Eng. 2025;37(8):4604–19. doi: 10.1109/tkde.2025.3573673

[47] Ma P, Ni Z. Few-shot time series forecasting in a meta-learning framework. IFS. 2024;46(4):8903–16. doi: 10.3233/jifs-233520

[48] Zhao W, Chen H, Yazan DM, Taghavifar H, Lyu Y, Bulis A. Few-shot learning and deep predictive models for cost optimization and carbon emission reduction in energy-water management. J Environ Manage. 2025;389:126077. doi: 10.1016/j.jenvman.2025.126077 40489924

[49] Ekambaram V, Jati A, Dayama P, Mukherjee S, Nguyen N, Gifford W, et al. Tiny Time Mixers (TTMs): Fast Pre-trained Models for Enhanced Zero/Few-Shot Forecasting of Multivariate Time Series. In: Advances in Neural Information Processing Systems 37, 2024. 74147–81. 10.52202/079017-2359

[50] Lian D, Zhou D, Feng J, Wang X. Scaling & Shifting Your Features: A New Baseline for Efficient Model Tuning. In: Advances in Neural Information Processing Systems 35, 2022. 109–23. 10.52202/068431-0009

[51] Zeng A, Chen M, Zhang L, Xu Q. Are Transformers Effective for Time Series Forecasting?. AAAI. 2023;37(9):11121–8. doi: 10.1609/aaai.v37i9.26317

[52] Zhou T, Ma Z, Wen Q, Wang X, Sun L, Jin R. Fedformer: Frequency enhanced decomposed transformer for long-term series forecasting. In: International conference on machine learning, 2022. 27268–86.

[53] Wu H, Xu J, Wang J, Long M. Autoformer: Decomposition transformers with auto-correlation for long-term series forecasting. Advances in Neural Information Processing Systems. 2021;34:22419–30.

[54] Zhou H, Zhang S, Peng J, Zhang S, Li J, Xiong H, et al. In: Proceedings of the AAAI Conference on Artificial Intelligence, 2021. 11106–15.

[55] Liu S, Yu H, Liao C, Li J, Lin W, Liu AX. Pyraformer: Low-complexity pyramidal attention for long-range time series modeling and forecasting. In: 2021.

[56] Li S, Jin X, Xuan Y, Zhou X, Chen W, Wang YX. Enhancing the locality and breaking the memory bottleneck of transformer on time series forecasting. Advances in Neural Information Processing Systems. 2019;32.

